# Critical thinking subskills in project-based EFL learning: uneven salience and conditional activation

**DOI:** 10.3389/fpsyg.2026.1750394

**Published:** 2026-04-07

**Authors:** Xue Hu, Shaidatul Akma Adi Kasuma, Feng Liu

**Affiliations:** 1 School of Languages, Literacies, and Translation, Universiti Sains Malaysia, George Town, Malaysia; 2 Jilin Engineering Normal University, Changchun, China

**Keywords:** cognitive processes, critical thinking, EFL secondary education, project-based learning, student perspectives

## Abstract

**Introduction:**

Most research on critical thinking (CT) in project-based learning (PBL) has focused on whether CT improves following instruction, typically through composite or dimension-specific scores. Less is known about how learners experience individual CT subskills during task engagement.

**Methods:**

This qualitative study examined how twelve Grade 12 students at a Sino-foreign cooperative secondary school in China described their use of six CT subskills during intercultural project-based tasks. Semi-structured interviews were conducted one week after task completion and analysed using a hybrid thematic approach.

**Results:**

In students’ accounts, the six subskills were not described as equally salient or simultaneously activated. Interpreting and analysing were commonly described as early, relatively consistent moves. Inferring and evaluating appeared more variable: under certain conditions they sharpened culturally responsive communication, while under others they narrowed cultural content through premature assumptions or receded into procedural compliance. Explaining and self-regulating were described as late-stage refinements, often compressed by time pressure. Perceived task stakes, rubric design, and feedback availability emerged as conditions that appeared to shape whether inference and evaluation took productive or constraining forms.

**Discussion:**

These findings suggest that CT subskills may be experienced as unevenly distributed across task phases, and that fostering CT in intercultural PBL may require explicit attention to the conditions under which less stable and later-emerging processes are supported.

## 1 Introduction

Critical thinking (CT) has become widely recognised as essential for learners navigating diverse perspectives, evaluating information from multiple sources, and adjusting their reasoning across linguistic and cultural boundaries (Abrami et al., 2015; Cash, 2017). In secondary English as a foreign language (EFL) contexts, where students are often preparing for international study, CT functions as a set of cognitive tools that enable them to process unfamiliar communicative situations, assess the appropriateness of linguistic choices, and reconsider their assumptions when engaging with culturally diverse audiences (Gómez-Rodríguez, 2018). When EFL tasks involve creating materials for international audiences, the cognitive demands on students intensify, as learners must interpret the backgrounds of their audience, evaluate whether information will be understood or misinterpreted, and adjust their explanatory strategies to bridge cultural gaps. In many Sino-foreign cooperative secondary schools in China, students participate in English-medium project tasks that simulate communication with international peers, making the deployment of CT in these tasks a key concern for pedagogy and research.

Project-based learning (PjBL) has been widely adopted as a pedagogical approach that can support CT development. Characterised by extended inquiry around authentic problems, collaborative decision-making, and the creation of tangible outcomes, PjBL is thought to create conditions under which students engage in active problem-solving, assume agency in planning their work, and present outcomes to audiences beyond the teacher (Markham et al., 2003; Thomas, 2000). Research has documented cognitive benefits associated with PjBL in EFL and related contexts, including improvements in problem-solving capacity, metacognitive awareness, and collaborative reasoning (Jalinus et al., 2020; Sudirman et al., 2023; Suteja and Setiawan, 2022). However, much of this evidence comes from quantitative designs using standardised tests or self-report measures to assess whether students’ CT improves following PjBL interventions (Abrami et al., 2015; Wu, 2011). While such studies have established that PjBL can support CT development when measured as overall test scores or general cognitive gains, they provide limited insight into how learners actually deploy specific CT subskills during project work, or whether certain subskills become more prominent depending on task characteristics. This outcome-focused orientation leaves the cognitive processes underlying reported gains largely opaque (Nguyen, 2021; Wu, 2020).

Among frameworks used to conceptualise CT, Facione (1990) model has proven particularly influential due to its operational clarity and empirical grounding. Derived from the American Philosophical Association’s Delphi project, the framework identifies six core CT subskills: interpretation (comprehending and expressing meaning), analysis (identifying relationships among concepts), inference (drawing reasonable conclusions), evaluation (assessing credibility and logical strength), explanation (stating and justifying reasoning), and self-regulation (monitoring one’s own thinking) (Facione, 1990). This framework has been operationalised in widely used assessment instruments and applied across multiple educational contexts. In EFL research, it has typically been used to measure CT as composite scores or dimension-specific subscales, with studies reporting whether overall ability or individual dimension scores increase following instruction (Indah et al., 2022; Moonma and Kaweera, 2022). This approach treats the six subskills as coequal and separable components (Algouzi et al., 2023; Indah et al., 2022) that contribute independently to CT performance. However, existing research on PjBL and CT has focused primarily on whether CT improves, rather than on how specific CT subskills are deployed during project work. To date, there is relatively little process-oriented evidence on how Facione’s six subskills are mobilised, sequenced, or constrained in authentic EFL tasks, or whether some subskills function as foundational moves while others emerge conditionally depending on task demands and perceived stakes (Nguyen, 2021; Wu, 2020).

Addressing this gap requires methodological approaches capable of capturing in-task cognitive processes. The present study employs a qualitative, narrative-based design, drawing on semi-structured interviews with secondary EFL learners to examine how they describe their own thought processes as they work through project tasks. The study was conducted with twelve Grade 12 students at a Sino-foreign cooperative school in China who had completed multiple project tasks with intercultural orientations over the course of their final academic year. By analysing how students narrated the cognitive work involved in creating materials for unfamiliar audiences, the study traces how they described using interpretation, analysis, inference, evaluation, explanation, and self-regulation when working on tasks that required them to interpret cultural information and construct outputs for international audiences. Rather than treating these subskills as parallel components, the analysis attends to how students positioned them as more or less central at different points in their project work.

The following research questions guide the study:

*RQ1:* How do secondary EFL learners describe deploying different CT subskills during project-based English tasks?

*RQ2:* Which CT subskills appear most salient across students’ narratives, and which appear less prominently?

*RQ3:* How do learners’ accounts illustrate the cognitive processes through which they interpret, select, and organise cultural information during project work?

This study contributes to research on CT and PjBL in EFL contexts by providing a task-based account of how learners described the unfolding of Facione’s six subskills during intercultural project work. It suggests that these subskills were experienced as a loosely ordered sequence, with interpretation and analysis anchoring early task engagement while other subskills emerged more contingently across tasks. The study also highlights conditions under which inference and evaluation appeared productive or problematic. Finally, it discusses practical implications for designing PjBL tasks and assessment criteria that may better support culturally reflexive CT in secondary EFL classrooms.

## 2 Literature review

### 2.1 Facione’s CT framework and its empirical operationalisation in EFL research

As outlined in the Introduction, Facione (1990) Delphi-based framework identifies six core CT subskills: Interpretation, Analysis, Evaluation, Inference, Explanation, and Self-regulation. The Delphi panel characterised these subskills as components of a single construct rather than as stages arranged in a fixed procedural sequence (Facione, 1990; Facione, 2015). This non-hierarchical characterisation has often been reflected in how CT is measured and reported across educational research (Halpern, 2014; Lai, 2011).

In EFL research, Facione’s framework has often been adopted as a measurement structure, but less often used as a lens for examining how subskills relate to one another during task engagement. Indah et al. (2022) assessed CT among Indonesian EFL students by reporting dimension-specific scores alongside an overall total. Algouzi et al. (2023) adopted a Facione-informed instrument in a Saudi EFL context and based their primary conclusions on pre-post changes in total CT scores, with subskill-level results receiving less analytic attention. In both cases, the subskills were primarily operationalised as scoreable dimensions, while their coordination during task engagement was not the main analytic focus. More broadly, EFL studies drawing on Facione’s framework have tended to focus on whether CT improves following instruction, rather than on how subskills are activated or coordinated within specific tasks (Nguyen, 2021; Wu, 2020). Such operationalisations are valuable for documenting CT attainment, but they reveal less about how subskills are experienced and coordinated while learners work through complex tasks.

Whether learners experience these subskills as equally salient, differently prioritised, or unevenly coordinated during authentic tasks remains largely underexplored. In EFL contexts, learners must engage in higher-order thinking through an additional linguistic medium, which may shape which subskills become more salient at different points in a task (Gómez-Rodríguez, 2018; Sobkowiak, 2016). This issue becomes particularly important in project-based learning, where tasks often require sustained inquiry, collaboration, and audience-oriented communication.

### 2.2 CT in PjBL research: from outcome gains to process questions

PjBL has often been associated with CT development because it engages students in sustained inquiry, collaborative decision-making, and the production of work for real audiences (Blumenfeld et al., 1991; Markham et al., 2003; Thomas, 2000). Meta-analytic evidence provides partial support for this association. Zhang and Ma (2023), synthesising 66 studies, reported a moderate positive effect of PjBL on thinking skills overall, though the effect on critical thinking specifically did not reach statistical significance. Abrami et al. (2015) identified dialogue and contextualised problem-solving as particularly effective strategies for CT, both central to PjBL design.

Several studies have reported positive effects. Sasson et al. (2018) observed a large effect on CT after two years of interdisciplinary PjBL in an Israeli secondary school. Song et al. (2025b) reported significant improvements in analyzing and evaluating skills following an 11-week online PjBL intervention with Chinese college EFL learners, with moderate to large effect sizes. Chiang and Lee (2016) found gains in motivation and problem-solving ability among PjBL participants. Other findings have been less consistent. Cash (2017) and Henderson et al. (2020) reported non-significant effects, and Xu et al. (2023) found that collaborative learning was more effective in developing CT dispositions than cognitive CT skills, suggesting that not all cognitive dimensions benefit equally from collaborative task formats.

A recurring limitation across these studies is their reliance on aggregate CT scores. Sasson et al. (2018) used a single composite measure. Most studies in Zhang and Ma (2023) meta-analysis employed instruments that did not differentiate among subskills (Tiruneh et al., 2017). This approach reveals whether PjBL improves CT as a whole, but cannot show which subskills are most responsive to project work or how they are activated across task phases.

Emerging work has begun to offer more differentiated accounts. Tola Chala et al. (2025) examined the effects of reflective writing on CT among Ethiopian EFL student-teachers and reported the largest gains for Analysis and Interpretation, with smaller effects for Evaluation, confirming that subskills can respond differentially to instructional conditions. Song et al. (2025a) used qualitative case study methods to investigate how PjBL fostered CT among Chinese college EFL learners at different proficiency levels. They identified four mechanisms through which PjBL supported CT: problem identification, information analysis, evaluative judgment through peer review, and reflective thinking. Wu (2011) similarly observed that Chinese EFL learners frequently employed metacognitive strategies during group project work, providing early evidence that CT-related cognitive engagement varies across task phases. These studies move beyond aggregate outcomes, yet they stop short of tracing how individual Facione subskills were differentially mobilised and coordinated within specific task episodes, and they do not examine CT through the complete six-subskill framework.

This gap is especially relevant in intercultural project tasks, where cognitive demands extend beyond general information processing. Tasks that require students to explain cultural practices to unfamiliar audiences involve audience-specific interpretation, selective content decisions shaped by anticipated misunderstanding, and evaluative judgments about cultural appropriateness (Byram, 1997; Gómez-Rodríguez, 2018). CT and intercultural communicative competence share overlapping cognitive skills, including analysis, interpretation, inference, and evaluation (Sobkowiak, 2016). Yet analyses of EFL textbooks have shown that cultural content rarely promotes independent critical reflection (Rezaee and Saleh, 2025). Empirical studies of intercultural tasks in EFL classrooms have reported gains in comparing cultural perspectives and challenging stereotypes (Esmaeili and Kuhi, 2023) and in identifying, interpreting, and evaluating cultural values (Susilo et al., 2023). These cognitive operations correspond closely to several of Facione’s subskills, yet the studies measured them as composite constructs rather than tracing how individual subskills were mobilised across task phases. Understanding how subskills are experienced and coordinated under such conditions requires approaches that go beyond aggregate scores and attend to learners’ accounts of their task engagement.

### 2.3 Qualitative and process-oriented perspectives on CT in task-based learning

Only a small body of work has adopted qualitative or process-oriented approaches to CT in language education. Most studies in this area have measured CT as an instructional outcome rather than examining how learners experience it during task completion.

Among the few exceptions, Liang and Fung (2021) traced how primary school ESL learners engaged in explicit reasoning and exploratory talk during group discussions. Their classroom observation data illuminated CT as it unfolded in interaction, demonstrating that process-oriented methods can capture dimensions of thinking that test scores cannot. The study focused on spontaneous dialogue rather than extended project work, and did not analyse CT through a subskill framework. Van Le and Chong (2024) interviewed Malaysian and Vietnamese undergraduates and identified a process-oriented conception of CT in which students described analysing information, evaluating ideas, and integrating perspectives as ongoing cognitive activities. Their findings confirm that learners can articulate CT as something they do rather than something they possess, though the study explored general perceptions rather than subskill mobilisation within specific tasks.

Related work has suggested that CT in language education is shaped by contextual, dialogic, and intercultural demands (Abrami et al., 2015; Lai, 2011; Wu, 2020). These claims, however, have more often been made at the level of theory or broader educational context than through fine-grained qualitative analyses of learners’ task engagement.

These studies indicate that qualitative approaches can illuminate how CT is experienced by learners during task work. To our knowledge, however, little qualitative research has examined how Facione’s six CT subskills are experienced, prioritised, and coordinated by EFL learners during intercultural project tasks. The present study addresses this gap by examining how EFL learners described the salience and coordination of CT subskills in intercultural project work.

## 3 Methods

### 3.1 Research design

This study adopts a qualitative, process-oriented exploratory design to examine how secondary EFL learners deploy critical thinking subskills during intercultural project-based tasks. Rather than measuring whether CT improves following instructional interventions, the study investigates how students describe their own thinking as they worked through tasks requiring them to construct explanations for unfamiliar international audiences. This approach is located within an interpretivist paradigm, treating students’ accounts as situated interpretations of their cognitive work rather than objective reports of mental processes. Semi-structured interviews were selected as the primary data collection method because this inquiry required access to learners’ subjective sense-making processes—how they articulated their reasoning, which cognitive moves felt most salient during task performance, and how they made decisions when interpreting and organizing cultural information for international audiences. It should be noted that the interview data reflect students’ retrospective accounts of their thinking rather than real-time records of cognition. The study therefore speaks to how learners perceived and reconstructed their cognitive processes, not to the exact processes that occurred during task performance.

### 3.2 Research context and participants

Twelve Grade 12 students from a Sino-foreign cooperative secondary school in China participated in this study. All participants had completed a sequence of five intercultural project-based tasks during their final academic year. All participants had studied English as a foreign language for at least ten years and had been enrolled in the school’s international curriculum track since Grade 10, where English served as a primary medium of instruction in preparation for overseas university study. By the time of data collection, they had experience completing project tasks that required English-language outputs. Interviews were conducted in Chinese to ensure that linguistic limitations did not constrain students’ ability to articulate complex reasoning processes. Students were recruited through purposeful sampling based on their demonstrated capacity to articulate reasoning processes during post-task reflections. This sampling strategy may have favoured students who were more comfortable articulating their reasoning; the findings should therefore be interpreted as representing the accounts of relatively articulate learners rather than the full range of CT engagement in this cohort.

During interviews, students were asked to recall one project task that remained particularly memorable and describe the thinking processes they had engaged in. Different students selected different tasks as most salient, resulting in natural variation across task types. This variation enabled examination of how CT subskills were deployed under different cognitive demands and task characteristics.

### 3.3 Project tasks

Students had completed five types of project-based tasks during their final academic year, all of which required them to construct materials for international audiences. Podcast involved recording audio episodes that introduced Chinese cultural topics, such as ancient inventions, Northeastern regional cuisine (e.g., guobaorou), or learning style differences between Chinese and foreign students. Idea Talk required creating presentations explaining Chinese secondary school routines, including morning reading sessions, evening self-study, dormitory systems, and class meetings, to incoming international students. Infographics involved designing visual comparisons of cultural practices, such as Chinese and foreign first-aid procedures, in accessible poster formats. Interactive Map required designing digital orientation materials with embedded QR codes providing English audio introductions to campus landmarks, cafeterias, and cultural heritage sites. Role-play simulated high-stakes scenarios in which students communicated with international dormitory supervisors during medical incidents such as allergic reactions, choking, burns, or broken bones, requiring rapid judgments about culturally appropriate actions and institutional protocols.

### 3.4 Data collection

Data were collected through semi-structured interviews conducted one week after the task was completed. This interval allowed students time to develop reflective distance from their work. At the same time, memories of their decision-making processes remained accessible. Nevertheless, retrospective accounts are susceptible to post-hoc rationalisation and should therefore be understood as students’ reconstructions of their thinking rather than direct evidence of in-task cognition. Interviews lasted between 30 and 40 min and were conducted in Chinese, enabling students to articulate complex reasoning processes in their native language. All interviews were audio-recorded, transcribed verbatim, and pseudonymised using codes S01 through S12.

The interview protocol asked students to recall one project task that remained particularly memorable and to describe the thinking processes they had engaged in during that task. Questions probed how students understood the task requirements and audience characteristics, which cognitive skills they deployed most prominently, how specific skills supported their work, and which skills they felt were less necessary or less suitable in that context. Follow-up prompts invited students to reconstruct specific decision-making moments, such as why certain content was included or deleted, how explanatory strategies were chosen, how tone or phrasing was adjusted before finalising their work, and how they weighed appropriateness or potential misunderstanding when making communicative choices. Facione’s six-dimensional CT framework informed these prompts. However, they remained open-ended to allow students to narrate their processes in their own terms. When students identified a particular cognitive skill as most helpful or least useful, interviewers asked them to explain the mechanisms through which that skill functioned, pressing for concrete examples from their task experience rather than abstract generalisations.

### 3.5 Data analysis

Transcripts were analysed using a hybrid thematic analysis that combined deductive coding informed by Facione’s (1990) framework with inductive attention to emergent patterns (Braun and Clarke, 2019; Fereday and Muir-Cochrane, 2006). While data were drawn from narrative interviews, the analytic focus was on identifying thematic patterns of CT deployment across cases rather than examining narrative structure per se.

All transcripts were read multiple times to identify segments where students explicitly commented on their thinking, using phrases such as “I realised,” “I decided to,” or “I was worried that.” Initial analytic notes highlighted moments when students described audience interpretation, content filtering, or tone adjustment. An initial coding frame based on Facione’s six CT subskills was then applied using NVivo 15. Six parent nodes corresponding to interpretation, analysis, inference, evaluation, explanation, and self-regulation were created. Segments describing audience understanding, information prioritization, anticipatory reasoning, appropriateness judgments, contextualization, and tone revision were coded accordingly. This deductive phase provided a structured starting point but did not constrain the analysis to these predefined categories.

Segments that did not fit neatly into Facione’s framework were coded inductively. Emergent codes captured patterns related to how students specified their target audience, the assumptions they made about audience knowledge or interests, the reasoning processes they employed in high-stakes scenarios, and the late-stage adjustments they made to linguistic tone. The coding frame was refined iteratively as analysis progressed. When overlaps or ambiguities arose, particularly between inference and evaluation, alternative categorisations were discussed, and the working coding frame was revised until a coherent distinction could be maintained in practice.

Comparisons were then made across different task types and different students to explore whether certain subskills consistently appeared as foundational moves while others functioned as late-stage refinements. NVivo node structures and code reports were used to track how categories were combined, separated, or renamed over time, providing a basic record of how the analysis evolved.

### 3.6 Trustworthiness

Following Guba and Lincoln (1994) criteria for trustworthiness in qualitative research, credibility, transferability, dependability, and confirmability were addressed in several ways. Credibility was established through thick description of students’ accounts, within-case and cross-case comparison, and collaborative coding discussions with a second researcher. Transferability was supported through rich contextual description of the research setting, participant profiles, and task design features. Dependability was addressed through maintaining an analytic log in NVivo documenting coding decisions and theme revisions, alongside iterative memo-writing that traced interpretive development. Confirmability was pursued through reflexive memos that prompted ongoing vigilance against selective interpretation, ensuring analysis remained grounded in students’ explicit statements.

### 3.7 Ethical considerations

This interview study formed part of a larger mixed-methods project on project-based learning, critical thinking, and intercultural communicative competence, which received approval from the university’s institutional ethics committee. All participants were 18 years of age or older at the time of data collection. Before interviews, informed consent was obtained from all student participants. Students were informed that participation was voluntary, that they could withdraw at any time without incurring an academic penalty, and that their identities would be protected through pseudonymization. All audio recordings and transcripts were securely stored and accessible only to the research team. Data were used solely for academic purposes, and no identifying information appears in published materials.

## 4 Results

### 4.1 Foundational moves: interpreting the task and analysing content

**Figure 1:**
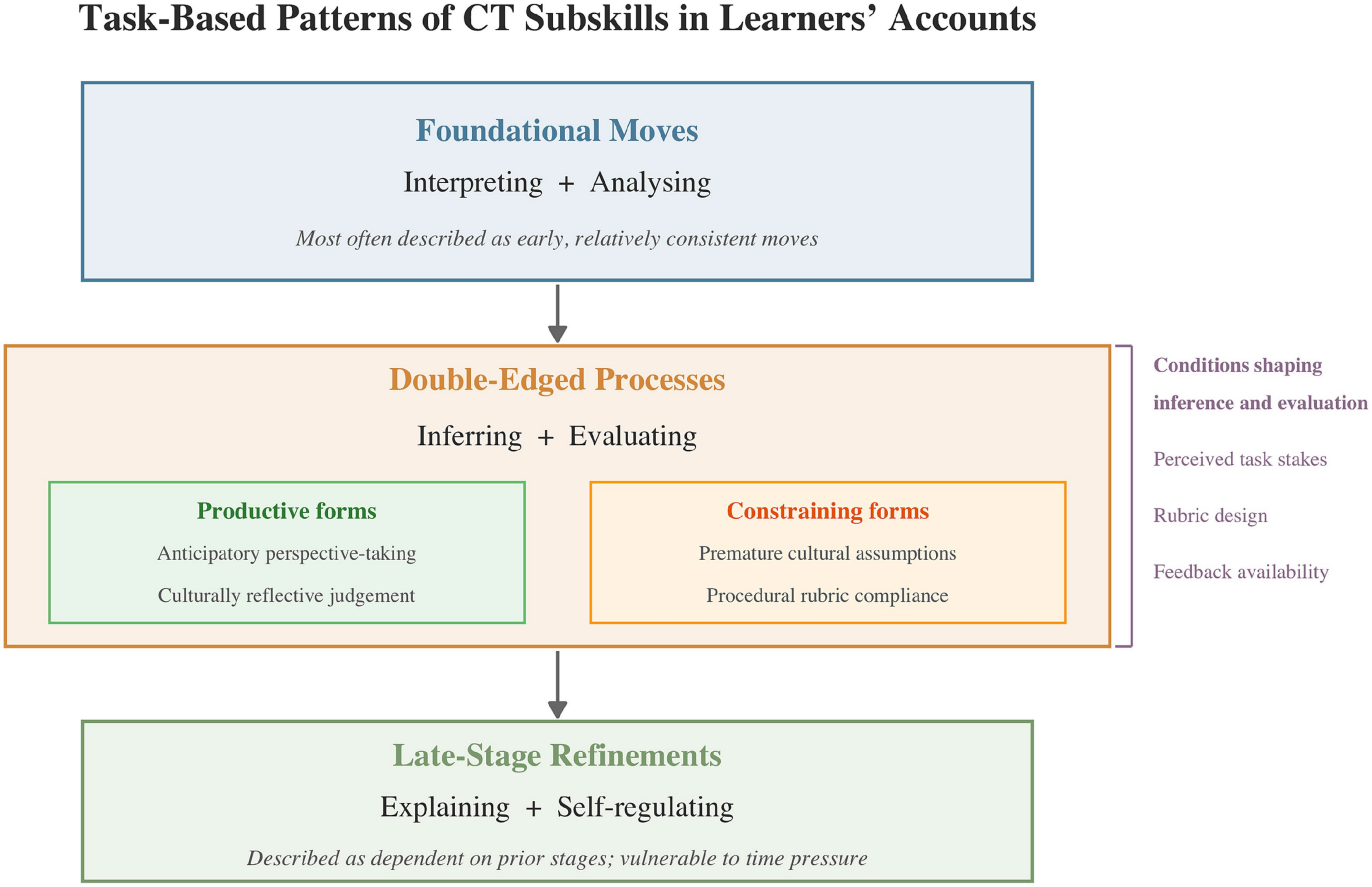
Task-based patterns of CT subskills as described in learners’ accounts.

The findings are organised around three functional clusters that reflect how students described the sequencing and salience of CT subskills across their project work, as illustrated in Figure 1.

Across the twelve accounts, interpreting and analysing were commonly described as the earliest cognitive moves. In practice, the two processes were closely linked. Students first formed a working sense of who the audience was and where misunderstanding might arise, then turned that understanding into decisions about what to prioritise and how to organise it. These processes constituted the foundation upon which later reasoning was built.

For most students, early task engagement began with identifying likely points of cultural mismatch between themselves and the intended audience. Students described actively scanning background materials to locate where international students might encounter confusion. One student recalled reading the teacher-provided article to “figure out why they come and what they struggle to adapt to” (S01, Idea Talk). A student working on the interactive map described a similar process of reading the same material to “figure out where they usually get stuck” (S03, Interactive Map). In both cases, students described interpretation as a way of locating anticipated gaps in the audience’s knowledge rather than simply comprehending the task brief.

In role-play tasks, this interpretive work extended further, to recognising differences in institutional and procedural frameworks. One student, simulating a medical emergency abroad, described needing to understand that she was operating within a foreign system: “I first knew this was abroad, in their dorm, under their rules” (S02, Role-play). This recognition reshaped what she chose to communicate, shifting her focus from describing the problem to explaining what had already been done within the host institution’s protocols. Other students drew on specific intercultural contrasts, such as differences in photo-taking permissions (S06) or first-aid customs like “pinching the philtrum” (S08). These contrasts sharpened their sense of what might be taken for granted at home but would require contextualisation for an international audience.

Students described this early interpretive work as shaping not only their content choices but also the communicative stance they adopted. One student explained that “once you understand the other party’s situation, you know what to say and how to say it” (S01, Idea Talk). Without this understanding, she added, “you might easily sound like you are lecturing them.” Another student noted that after reading about international students’ common frustrations, she began to frame unfamiliar practices as “common in Chinese schools” rather than presenting them as rules requiring compliance (S03, Interactive Map). The interpretive process enabled a shift from an instructional register to one that acknowledged difference without imposing judgment. Accounts in which this foundation appeared less fully developed also contained more uncertainty about later decisions, particularly how much explanation the audience would need.

Once a working understanding of the audience was in place, students described shifting into analytic work: deciding what to include, what to leave out, and how to structure their output. This filtering process was especially prominent in content-rich tasks. A student creating a comparative infographic recalled the challenge of working through a large volume of material: “You had to dig out the most essential stuff that could be compared” (S08, Infographic). Another framed the same process in explicitly audience-centred terms, asking what “visiting students most want to know and what’s most useful” (S06, Idea Talk). Analysis also extended to sequencing. A student preparing for a role-play emergency described organising her communication deliberately: “I’d say the location first, then the symptoms, then what we’d already done” (S02, Role-play). She characterised this as “thinking through how to say it without getting confused.” In this account, deciding what to say first served simultaneously as a way of managing the communicative demands of the situation.

Although students commonly described interpretation and analytic filtering in close succession, the boundary between the two processes was not always sharply drawn. One student characterised her early work on a podcast as “analysing how foreign students might think about us” (S04, Podcast), a formulation that blended audience interpretation with evaluative comparison. In tasks where the audience was more abstractly defined, interpretation sometimes merged with analysis rather than clearly preceding it. These variations suggest that the sequencing of the two processes was shaped in part by the specificity of the audience description and the contextual materials the teacher provided. With this interpretive and analytic foundation in place, students moved into less predictable cognitive territory. The processes of inferring and evaluating required them to move beyond understanding and selection toward more anticipatory and evaluative judgments. These later judgments were described as less stable across task contexts.

### 4.2 Double-edged processes: inferring and evaluating across task contexts

Where interpreting and analysing were described as relatively stable across tasks, inference and evaluation occupied less predictable ground. Students’ accounts portrayed these two processes as capable of either strengthening or weakening their communicative choices, depending on the conditions under which they were carried out. What made these processes distinctive was not their presence or absence but the direction they took: under certain conditions, they sharpened students’ responsiveness to the audience; under others, they narrowed the cultural content students chose to communicate or reduced evaluation to procedural checking.

In its more productive form, inference enabled students to anticipate where an audience might encounter confusion or misunderstanding. This anticipatory work was described across several task types. One student recalled guessing that international students would have no idea what “morning reading” was, and planned additional explanation accordingly (S01, Idea Talk). Another estimated that a foreign dorm supervisor would not understand the Chinese practice of informing parents first, and prepared what to say in advance (S02, Role-play). A third described asking herself, “What would a foreigner who’s never been to our school most want to know?” and characterised this anticipatory work as “a kind of invisible respect” (S09, Interactive Map). In these accounts, inference functioned as a form of perspective-taking that allowed students to prepare for gaps in shared knowledge before communication occurred.

Evaluation took on a similarly substantive character in tasks where students perceived their actions as carrying real consequences. This pattern was most visible in role-play scenarios. One student described shifting from an initial belief that “with first aid, as long as it’s fast, it’s fine” to recognising that she “also had to consider the other person’s cultural habits” (S05, Role-play). Another articulated the evaluative process as “quickly weighing in your mind: is what I’m doing appropriate? Will this be offensive or culturally inappropriate?” (S10, Role-play). A third came to see respecting “other people’s cultural customs” as “also part of saving them” (S12, Role-play). Outside role-play, a student working on a visual infographic applied similar reasoning, asking herself whether her cultural comparisons might be perceived as biased (S08, Infographic). What these accounts shared was a sense that evaluation, when prompted by perceived consequences, moved beyond checking correctness toward weighing appropriateness.

A different pattern emerged when inference was carried out with high confidence but limited grounding. Several students described making strong predictions about what the audience would or would not find interesting, and then using those predictions to delete content. One student reflected that she had “overused inference,” having removed material about evening self-study because she “kept thinking to myself that foreign students definitely would not be interested” (S01, Idea Talk). The teacher’s feedback later indicated that this content effectively conveyed cultural differences. Another student nearly reduced the school history museum to a single photograph, reasoning that “foreigners would definitely find it boring” (S09, Interactive Map). A simulated feedback session later revealed genuine curiosity about that content. She reflected: “I almost overlooked our school’s core cultural heritage because of my own ‘taken-for-granted’ assumption. This inference thing, when used well, it’s considerate, but when overused, it’s like looking at people through tinted glasses” (S09, Interactive Map). A third student added extensive food delivery options based on inferred audience interests, only to be told this mismatched the task’s focus (S03, Interactive Map). In these cases, the repeated use of “definitely” suggested that inferences were being treated as certainties rather than tentative guesses. In several accounts, no external feedback was described as having challenged these certainties.

Under lower-stakes conditions, evaluation followed a parallel pattern of narrowing. Students working on presentations, maps, and podcasts more often described evaluation in procedural terms. One student reported that her evaluation “was basically just checking against the teacher’s rubric” (S01, Idea Talk). Another acknowledged that her group’s evaluation amounted to “going through the motions,” with members agreeing that work was “more or less okay” without deeper scrutiny (S08, Infographic). She attributed this partly to time pressure and partly to the recognition that “this wasn’t for real foreigners to see, it’s just an assignment.” In these accounts, evaluation did not engage with cultural content, audience perception, or the risk of misunderstanding. It focused instead on compliance with rubric specifications.

What distinguished the productive pattern from the constraining one was less whether students inferred or evaluated than how, and under what conditions, they did so. When students framed their inferences as questions or possibilities, they tended to use them to add context or pre-empt confusion. When they treated inferences as settled facts, they were more likely to remove content that later proved valuable. Similarly, when students perceived the task as carrying real consequences, evaluation became more active and culturally engaged. When the task felt hypothetical or when rubrics foregrounded technical features over cultural appropriateness, evaluation receded into routine compliance. The availability of feedback also played a role. Students whose inferences were later challenged, whether by teachers or through simulated audience responses, recognised the gap between their predictions and the audience’s actual interests. Students who received no such feedback described their inferences as going unchecked.

Not every account fitted neatly into these two patterns. Some students described inference and evaluation only briefly, without elaborating on the conditions that shaped them. The distinction between productive and constraining forms was clearest among students who had received feedback or who reflected on moments where their initial judgments proved inaccurate. For students who did not encounter such moments, the two modes were less clearly differentiated in their accounts. With inference and evaluation described as contingent and variable, the remaining two processes, explanation and self-regulation, occupied a different position in students’ accounts. These were described less as moments of judgment than as late-stage refinements carried out under practical constraints.

### 4.3 Late-stage refinements: explaining and self-regulating under constraint

Explanation and self-regulation were commonly described as appearing toward the end of the task process, after interpretive and analytic groundwork had been laid and after key content decisions had already been made. Rather than independent cognitive operations, students described these processes as refinements of material that had already been gathered, filtered, and organised. Both were also described as vulnerable to time pressure, fatigue, and competing demands from group collaboration.

Explanation involved making culturally embedded practices intelligible to an audience presumed not to share the same background knowledge. One student, creating an interactive map, described adding context to unfamiliar school routines: when introducing “class meetings,” she explained that they were “for building class spirit,” because she “was worried they might think it was the teacher criticizing students” (S03, Interactive Map). Another described needing to explain why she had contacted the dormitory’s emergency desk rather than dialling 110, adding “this is the procedure for international students” to pre-empt the impression that she was delaying help (S02, Role-play). In both cases, students described explanation as a way of articulating the rationale behind practices that might otherwise appear arbitrary or inappropriate to an outside audience.

Explanation also involved adjusting how content was communicated linguistically. One student described this as learning to “translate” formal language into plain speech, noting that when thinking about explaining to an audience, “you automatically replace those words that might sound arrogant or unclear” (S07, Podcast). Another intervened when a group member “kept using those really formal bookish sentences,” reasoning that “foreign people will not understand this stuff” (S04, Podcast). In a few accounts, explaining also prompted students to refine their own understanding of the material. One student, preparing a podcast about a regional dish, discovered through background research that it had originated as a dish designed to suit Russian guests’ tastes, and reflected: “I used to think of it as a dish. Now I understand that from birth it was an intercultural product” (S11, Podcast). Although less commonly described, this account showed that explanation could, under certain conditions, move beyond packaging existing knowledge.

Self-regulation appeared less frequently in students’ accounts and was typically described as a final checking stage rather than an integrated part of the working process. The most common form involved revising tone to soften directness or reduce imperative language. One student described reviewing her map descriptions before submission, “changing things like ‘you must’ to ‘you can’” (S03, Interactive Map). Another revised evaluative language in a podcast script, replacing a comparative judgment with the more neutral formulation “learning styles are somewhat different” (S04, Podcast). These revisions reflected an awareness that directness norms varied across cultures. However, students also described self-regulation as constrained by practical circumstances. One student noted that it “helped improve the attitude a bit” but “wasn’t as core as interpretation” (S01, Idea Talk). Others described being “exhausted” by this stage and approving work that was “more or less okay” without further revision (S07; S09).

The accounts of explanation and self-regulation shared a common feature: both were described as dependent on prior stages having been completed. Where interpretation and analysis had produced a clear sense of audience and content, explanation could focus on contextualisation and register. Where earlier stages had been rushed, students described explanation as more uncertain and self-regulation as more perfunctory. Not all students described these processes with equal detail. Self-regulation in particular received brief treatment in several accounts, with some students acknowledging that they had “cut corners” at this stage (S09, Interactive Map). Compared with the fuller descriptions of earlier processes, these accounts were relatively thin. This was consistent with students’ tendency to describe explanation and self-regulation as later, less elaborated, and more practically constrained stages of their task work.

## 5 Discussion

### 5.1 Revisiting Facione’s framework through task-based learner accounts

Facione’s (1990) Delphi-based framework characterises the six CT subskills as components of a single construct rather than as stages arranged in a fixed procedural sequence (Facione, 2015). This non-hierarchical characterisation has shaped how CT is typically measured in educational research. Empirical studies in EFL contexts have generally operationalised the subskills as parallel scoreable dimensions, reporting composite or dimension-specific scores without examining how the subskills relate to one another during task engagement. The present findings add an experiential dimension to this picture. In students’ accounts of their project work, the six subskills were not described as equally salient or simultaneously activated. Instead, learners described a loosely ordered pattern in which interpreting and analysing appeared earlier and more consistently, while the remaining subskills were described as more contingent and more readily compressed under task constraints.

This pattern does not contradict Facione’s framework, which was not designed to specify a temporal order. It does, however, suggest that when learners engage with complex intercultural tasks, they may experience the subskills as unevenly distributed across the task process, with some becoming more salient at particular stages than others. Recent quantitative work has pointed in a similar direction. Tola Chala et al. (2025) reported that reflective writing produced the largest gains for Analysis and Interpretation among Ethiopian EFL student-teachers, with smaller effects for Evaluation. Song et al. (2025b), using Paul and Elder (2020) framework, found that online PBL significantly improved analyzing skills among Chinese college EFL learners, while evaluating skills showed only marginal change. Although these studies measured CT as an instructional outcome rather than tracing subskill coordination during tasks, their findings are consistent with the present study’s observation that not all subskills were described as equally prominent or equally responsive to task demands.

Qualitative research has begun to explore CT as a process rather than a measured outcome, though few studies have done so at the level of individual subskills. Van Le and Chong (2024) found that university students described CT as ongoing cognitive activities, including analysing, evaluating, and integrating perspectives, rather than as a fixed ability. Their findings confirm that learners can articulate CT in process terms, but the study did not trace how these activities were sequenced or prioritised within specific tasks. Song et al. (2025a) identified four mechanisms through which PBL supported CT among Chinese college EFL learners, including problem identification, information analysis, evaluative judgment through peer review, and reflective thinking. These mechanisms resonate with several of the patterns described in the present study, particularly the foundational role of interpretation and analysis and the contingent character of evaluation. However, Song et al. (2025a) did not map these mechanisms onto a subskill framework or examine their relative prominence across task phases. The present study contributes to this emerging line of work by showing that, in learners’ accounts, the six Facione subskills were experienced as differentially salient, with some appearing earlier and more consistently than others across task phases.

Viewing CT through learners’ task-based accounts, rather than through aggregate test scores, brings into focus a dimension that outcome-focused studies cannot easily capture: the uneven salience of subskills across different stages and conditions of task engagement. The next section considers one set of these conditions in more detail, by examining the conditions under which inference and evaluation took productive or constraining forms.

### 5.2 Inference and evaluation as sites of productive tension between CT and ICC

Of the six subskills examined in this study, inference and evaluation showed the widest variation in how students described using them. As reported in the findings, these processes took on either productive or constraining forms depending on the conditions under which they were carried out. This variability is theoretically significant because it points to a set of conditions under which CT processes may either support or work against the goals of intercultural communicative competence. Where CT and ICC have often been discussed as complementary, the present findings suggest that their relationship is more conditional than typically assumed.

In their productive forms, inference and evaluation enabled students to engage in what can be understood as anticipatory perspective-taking and culturally reflective judgment. Students who framed their inferences as questions or possibilities described using them to pre-empt audience confusion and to add contextual explanation before miscommunication occurred. In high-stakes tasks, evaluation prompted students to weigh cultural appropriateness alongside technical effectiveness, holding competing criteria in mind simultaneously. These forms of reasoning align with what Byram (1997) terms critical cultural awareness: the capacity to evaluate practices and products in one’s own and other cultures using explicit criteria. They also correspond to the attitudinal foundations of Deardorff (2006) ICC model, in which openness, respect, and a willingness to withhold premature judgment are described as preconditions for effective intercultural engagement. In these accounts, CT subskills and ICC dispositions appeared to work in tandem, with inference and evaluation serving as the cognitive means through which culturally sensitive communication was pursued.

In their constraining forms, however, the same processes moved in the opposite direction. When students treated their inferences as settled facts rather than tentative predictions, their audience reasoning narrowed. Content that might have conveyed cultural richness was removed on the basis of confident but untested assumptions about what the audience would find uninteresting or irrelevant. This pattern represents a form of premature cultural assumption in which tentative reasoning hardened into certainty in the absence of external feedback. Facione (2015) identifies truth-seeking and open-mindedness as dispositions that moderate the quality of CT performance. The constraining pattern observed in this study suggests that when these dispositions were not actively supported, inference could slip from anticipatory perspective-taking toward overgeneralised expectation. Similarly, when evaluation was carried out under lower-stakes conditions or when rubrics foregrounded technical features, it receded into procedural compliance rather than engaging with cultural content. Sobkowiak (2016) has noted that CT and ICC share overlapping cognitive skills, including analysis, interpretation, inference, and evaluation. The present findings add a qualification to this observation: the overlap between CT and ICC may depend not only on which skills are activated but on the conditions under which they are exercised.

These findings carry a broader implication for how the relationship between CT and ICC is understood in EFL education. Much of the existing literature treats CT as inherently supportive of intercultural learning. Empirical studies of intercultural tasks in EFL classrooms have reported gains in comparing cultural perspectives (Esmaeili and Kuhi, 2023) and in identifying and evaluating cultural values (Susilo et al., 2023), and these outcomes are often attributed to the activation of CT processes. Yet analyses of EFL textbooks have shown that cultural content alone rarely promotes independent critical reflection (Rezaee and Saleh, 2025). The present study extends this concern from materials to learner cognition. When inference operates efficiently but without reflective checks, it may produce communicative choices that are swift but culturally narrowing. In other words, thinking that appears efficient from a task-completion perspective may, under certain conditions, function as a form of premature closure that reduces rather than enriches intercultural content. This possibility suggests that fostering CT in intercultural EFL contexts requires attention not only to whether students infer and evaluate but to the epistemic quality of those processes.

The conditions that shaped whether inference and evaluation took productive or constraining forms were not limited to individual dispositions. Task design, perceived stakes, and the availability of feedback all appeared to play a mediating role. The following section examines these structural and pedagogical conditions in more detail.

### 5.3 Task conditions, assessment design, and the limits of simulated authenticity

The findings reported in this study point to three conditions that appeared to shape whether inference and evaluation took more productive or more constraining forms: the perceived stakes of the task, the design of assessment criteria, and the availability of feedback.

The first condition concerned the perceived consequences of the task. Students’ accounts suggested that evaluation became more substantive when the task context made cultural missteps feel consequential. In role-play scenarios, the simulated risk of causing offence or physical harm prompted students to weigh competing criteria, balancing urgency against cultural respect. In tasks that students perceived as hypothetical classroom exercises, evaluation tended to recede into rubric compliance. This pattern suggests that it was not the presence of a real audience but the salience of potential consequences that activated more reflective evaluative thinking. PBL is widely recognised for creating meaningful and engaging learning experiences (Almulla, 2020), and meta-analytic evidence supports its association with improved thinking skills (Zhang and Ma, 2023). Yet the present findings suggest that engagement and authenticity alone may not be sufficient to sustain deeper forms of CT. What appeared to matter was whether the task design made the consequences of cultural misjudgment feel tangible to students.

The second condition concerned the design of assessment criteria. Several students described evaluating their work primarily against technical features such as image clarity, grammatical accuracy, or slide count, without attending to cultural appropriateness or audience perception. This pattern raises questions about whether commonly used assessment rubrics may be misaligned with how CT subskills are actually experienced during task work. Many CT assessment instruments operationalise the subskills as parallel, simultaneously scoreable dimensions (Tiruneh et al., 2017), and PBL studies have often relied on composite CT measures that do not differentiate among subskills (Sasson et al., 2018; Zhang and Ma, 2023). If, as the present findings suggest, students experience these subskills as unevenly distributed across task phases, then assessment designs that treat subskills as separately scoreable at a single point in time may overlook the uneven and compressed character of later-stage processes such as evaluation and self-regulation. This does not mean that existing rubrics are invalid, but it does suggest that rubric design may benefit from attending to the temporal and conditional dimensions of CT engagement, for instance by including criteria that explicitly name cultural appropriateness and audience responsiveness as assessable qualities.

The third condition concerned the availability of feedback. Students whose inferences were later challenged, whether by teachers or through simulated audience responses, described recognising the gap between their predictions and the audience’s actual interests. Students who received no such feedback described their inferences as going unchecked. This finding is consistent with research emphasising the role of dialogue and structured interaction in CT development (Abrami et al., 2015; Abrami et al., 2008). In the present study, feedback functioned not as a correction mechanism but as a condition that allowed students to treat their inferences as revisable rather than settled. Wu (2011) found that Chinese EFL learners frequently employed metacognitive strategies during group project work. The present findings suggest, however, that such metacognitive engagement may not extend to sustained self-monitoring of inference quality when feedback structures are absent and when the task feels low-stakes. Building structured feedback moments into PBL designs, whether through peer review, guided reflection, or engagement with external audiences, may help interrupt the self-confirming quality of unchecked inference (Bowen and Peterson, 2018).

These three conditions, perceived stakes, rubric design, and feedback availability, operated at the level of task and instructional design rather than at the level of individual learner ability. This observation carries practical implications for how PBL tasks are structured and assessed in intercultural EFL classrooms. The following section outlines these implications in more specific terms.

### 5.4 Implications for integrating CT and ICC in secondary EFL classrooms

The loosely ordered and contingent character of how students described engaging with CT subskills points to several considerations for secondary EFL teachers seeking to integrate critical thinking with intercultural learning. These implications follow from the three conditions discussed in the preceding section and are offered as design principles rather than prescriptive recommendations.

First, making audience specificity explicit from the outset may strengthen the interpretive foundation and reduce the risk of premature inference. Students in this study suggested that clearer audience definitions would have helped them direct their inferences toward anticipating audience questions rather than toward deleting content preemptively. This aligns with research emphasising that contextualised problems and clear task parameters support more focused critical thinking (Abrami et al., 2015). Second, incorporating peer or external feedback loops may interrupt the self-confirming quality of unchecked inference and create conditions for more substantive evaluation. Students themselves proposed connecting with international peers who could respond to drafts before submission. Even where direct access to international audiences is not feasible, structured peer review protocols in which students adopt the perspective of the target audience could serve a comparable function. Song et al. (2025a) identified peer review as one of four mechanisms through which PBL supported CT among Chinese college EFL learners, lending empirical support to this suggestion.

Third, providing cultural case examples that illustrate how misunderstandings arise may make the stakes of inference and evaluation more tangible in lower-risk tasks. Students indicated that exposure to real instances of communication failure across cultures would have given them more concrete grounds for evaluative judgment. This is consistent with evidence that dialogue and contextualised problem-solving are particularly effective strategies for CT development (Abrami et al., 2015; Abrami et al., 2008; Sudirman et al., 2023). Fourth, redesigning assessment rubrics to include cultural appropriateness as an explicit and weighted criterion may help elevate evaluation beyond procedural checking. As discussed in the preceding section, rubrics that emphasise technical features without naming cultural sensitivity or audience responsiveness may inadvertently signal that these dimensions are secondary. Making cultural criteria explicit and allocating meaningful weight to them in grading could help signal that reflexive evaluation is a central rather than optional expectation.

Finally, scaffolding inference and evaluation through guided questions may help students articulate and examine their assumptions at points during the task cycle when these processes are most vulnerable to compression. Wu (2011) found that metacognitive strategies were frequently activated during group project work among Chinese EFL learners, suggesting that explicit prompts for reflection can foster deeper engagement when built into the task structure rather than appended at the end. These implications caution against assuming that CT and ICC will be fostered simply because PBL tasks involve intercultural content. The development of both depends on instructional choices that make cultural dimensions visible, create structures that test assumptions, and scaffold evaluative criteria that students may not generate independently. While the present study focused on Chinese secondary EFL learners preparing for overseas study, these design principles may have relevance in other contexts where learners are asked to communicate across cultural or linguistic boundaries.

## 6 Conclusion

This study examined how secondary EFL learners described their use of critical thinking subskills during intercultural project-based tasks. By analysing students’ retrospective accounts through Facione’s (1990) six-subskill framework, the study offered a task-based perspective on CT that complements the outcome-focused emphasis of much existing research. The findings suggested that learners did not experience the six subskills as equally salient or simultaneously activated. Instead, interpreting and analysing were described as foundational to early task engagement, while inferring and evaluating appeared more contingent and variable, and explaining and self-regulating were described as late-stage refinements vulnerable to practical constraints. The study further identified perceived task stakes, rubric design, and feedback availability as conditions that appeared to shape whether inference and evaluation took productive or constraining forms. These observations contribute to an understanding of CT not only as a measurable outcome but as a set of processes that learners experience as unevenly distributed across the phases and conditions of task work.

Several limitations should be acknowledged. The study relied on retrospective interviews conducted one week after task completion, and the data therefore represent students’ reconstructions of their thinking rather than real-time records of cognition. Purposeful sampling based on demonstrated articulateness may have favoured more reflective learners, and the findings should be interpreted accordingly. All participants came from a single Sino-foreign cooperative secondary school, which limits transferability to other institutional and cultural contexts. The study also did not collect student artifacts such as completed projects or written outputs, meaning the findings speak to perceived cognitive processes rather than to the quality of the work produced.

Future research could extend this work in several directions. Longitudinal and multi-site designs would help establish whether the patterns observed here are stable across different tasks, learner populations, and curricular contexts. Incorporating real-time data collection methods such as think-aloud protocols or classroom observation, alongside the analysis of student artifacts, would allow for triangulation between how students describe their thinking and what their completed work reveals. Finally, studies that experimentally vary perceived stakes, rubric design, and feedback timing could test whether these conditions causally influence the depth and direction of CT subskill engagement in PBL settings.

Despite these limitations, the study offers a detailed account of how EFL learners described experiencing CT as a set of unevenly distributed and conditionally activated processes during intercultural project work. This perspective may inform the design of PBL tasks and assessment criteria that attend not only to whether critical thinking occurs but to when, how, and under what conditions it is sustained.

## Data Availability

The raw data supporting the conclusions of this article will be made available by the authors, without undue reservation.
